# Influence of school-related factors on smoking among Chilean adolescents: a cross-sectional multilevel study

**DOI:** 10.1186/s12887-016-0612-z

**Published:** 2016-06-09

**Authors:** Jorge Gaete, Catalina Ortúzar, Pedro Zitko, Alan Montgomery, Ricardo Araya

**Affiliations:** Department of Public Health and Epidemiology, Faculty of Medicine, Universidad de los Andes, Monseñor Álvaro del Portillo 12455, Las Condes, Santiago, Chile; Department of Population Health, London School of Hygiene and Tropical Medicine (LSHTM), Keppel Street, London, WC1E 7HT United Kingdom; Pontificia Universidad Católica de Chile, Av. Vicuña Mackenna 4860, San Joaquín, Santiago, Chile; Unidad de Estudios Asistenciales, Complejo Asistencial Barros Luco, José Miguel Carrera 3604, San Miguel, Santiago, Chile; Medical Statistics and Clinical Trials, Nottingham Clinical Trials Unit, University of Nottingham, C Floor, South Block, Queen’s Medical Centre, Nottingham, NG7 2UH United Kingdom

**Keywords:** Smoking, Adolescent health, School effect, School achievement, School truancy

## Abstract

**Background:**

Adolescent tobacco smoking is a major health concern in Chile. Schools may be able to influence adolescent behaviour regarding smoking; however, this topic has received limited research attention in Latin-American countries. Moreover, the prevalence of cigarette smoking varies between schools, and some of this variability may be explained by school factors. This article examines the inter-school variability in student smoking in a large sample of Chilean schools and identifies the school- and student-level characteristics associated with cigarette smoking.

**Methods:**

This cross-sectional study used self-reported student-level data from 45,273 students from 1462 schools and official data from these schools provided by the Chilean Ministry of Education (2007). Student smoking behaviour was used as an outcome, and individual-level and school-level features were used as explanatory variables. Logistic multilevel modelling was used to analyse the data.

**Results:**

The mean prevalence of smoking in the 1462 schools was 39.9 %. The null model indicated that 8 % of the variance in smoking behaviour was explained by schools; and in the final model, controlled by individual- and school-level variables, the variance explained by schools dropped to 2.4 %. The main school-level variables explaining the school influence were school bonding, school truancy and school achievement.

**Conclusions:**

This is the first study to examine the extent to which student smoking varies between Chilean schools and to identify some of the school factors associated with this inter-school variability. Although most variation in smoking prevalence lies between students within schools, there is sufficient between-school variation to be of interest to educators and policy makers.

## Background

Cigarette smoking among adolescents is a public health problem [1, 2]. Most of the adults with nicotine use disorder start nicotine use in their adolescent years [3]. Cross-country comparison studies have shown that Chile has the highest prevalence of cigarette smoking among students in the world. For instance, one study, which included 44 countries, showed that the monthly smoking prevalence was 32.8 %, higher than in most other countries [4]. Another report [5] from 43 countries showed that the median rate for current monthly smoking was 13.9 %, and the highest rate was found in Coquimbo, Chile (39.6 %). Although Chilean official figures show that smoking behaviour has been decreasing in recent years [6], it remains a major problem and its causes are not completely understood.

Different risks and protective factors related to smoking have been described among adolescents in the last decades [7]. These factors manifest themselves at different levels (e.g., personal, familial, school level) introducing complexity when trying to understand the behaviour of adolescents.

At a personal level, the prevalence of smoking among adolescents rises with increasing age [8]. Male youngsters smoke in a higher proportion than females [9, 10]. However, this gender difference seems to be decreasing [11], especially in high- [12] and middle-high income countries such as Chile and Brazil [11, 13]. With respect to emotional status, depression is a well-established risk factor for smoking in adolescents [14]. Unsurprisingly, adolescents with a positive attitude towards smoking [15] and those less aware of health risks associated with smoking seem more likely to smoke [14]. Finally, the amount of pocket money the adolescents receive has been associated with increased risk of smoking [16].

Recent reviews have suggested that several family factors can influence tobacco use [17, 18]. First, smoking within the family (parent or siblings) is associated with an increased risk of smoking among adolescents but the evidence is still limited and inconsistent [18]. In Chile, two studies have found an association between parental and adolescent smoking [19, 20]. There is also evidence from elsewhere that other familial features, such as good communication and positive relationships among family members, higher parental monitoring [21], stronger family attachment [17], higher parental support, and positive parenting style [22] might be protective factors against adolescent smoking.

As far as school factors are concerned, it is important to distinguish between individual (students) and contextual (school) influences. Studies analysing individual data have found that increased risk for smoking is associated with poor academic performance, low educational aspirations and low school commitment [21, 23], school disengagement and poor teacher-student relationships [24], and school smoking restrictions were effective, but only if they were appropriately enforced [25].

Several authors have found significant intra-school correlations in smoking onset, monthly smoking prevalence and the number of cigarette smoked per day [26–29], which could be related to the characteristics of the students within each school or other school contextual features [30]. Those studies exploring contextual effects using multilevel modelling have found that schools with a combination of higher performance and less truancy [31], schools receiving social assistance [32] and mixed sex or vocational high schools had a higher risk for smoking [33].

However, no multilevel study has explored the association between school bonding, at school level, and smoking, controlling for several individual and school-related variables. School bonding refers to the relationship between students and their schools (e.g. liking the school, feeling part of the school and having good relationships with their teachers) [34]. The aims of this study are: (i) to assess inter-school variation in smoking prevalence in a sample of Chilean schools and; (ii) to determine if school-level variables, such as school bonding, may explain some of the variation on smoking behaviour.

## Methods

### Participants

This study gathered information at two levels: students and schools. Individual-level information comes from 8th to 12th graders attending municipal, subsidised and private Chilean schools that participated in the 7th School Survey of Substance Use, conducted by the National Service for the Prevention and Rehabilitation of Drugs and Alcohol abuse (SENDA, former National Council for Narcotics Control -CONACE) in 2007. School-level information came from three sources: i) School registry from the Chilean Ministry of Education (2007), ii) School registry of the National System of the Measurement of the Quality of Education (SIMCE) from 2007 and 2008, and iii) aggregated variables at the school-level from students’ answers in the 7th School Survey of Substance Use.

The Chilean School Survey of Substance Use is a nationally representative survey conducted every two years since 1999. Each time, the survey gathers information about substance use and related factors. These risk factors are not measured every time. For example, items regarding school bonding and school climate were measured in 2007 the last time. Newer surveys have limited the number of items measuring school factors. The 7th School Survey of Substance Use is based on a nationally representative sample of 52,145 students from 3,048 classes attending 1,512 schools. From each class, 20 students were randomly selected. This sample represents 968,996 students from 86 cities in Chile with 95 % confidence and 4 % sampling error. Students answered a self-reported questionnaire in their classrooms.

The SIMCE dataset included results from math and language standardised tests for all schools in Chile. Year 2007 gathered information from 4th and 8th grades. Year 2008 gathered information from 8th and 10th grades. For the purposes of this study, both years were included. Because not all schools have all grades (for example, there are Chilean schools that have Years 1 to 8, and others that have Years 9 to 12), including the results from just one year could have dropped many schools from the analyses. Moreover, there is almost no variation in the results from one year to the next.

Finally, the 2007 School registry from the Chilean Ministry of Education provided information about schools such as the type of school and school sex composition.

### Variables

#### Outcome: monthly smoking

Monthly smoking referred to having smoked at least one day in the last 30 days before the survey. It was a binary variable. This is one of the most frequently used measures of current smoking, which allows us to compare our results to other studies [4, 5, 35].

#### Individual-level variables (Level 1)

Personal and family variables were included (see Table 1 for descriptive features of individual-level variables). Following recommendations from Aveyard et al. [36], we excluded all variables used to create school-level variables at this level, such as school bonding and truancy perception. Personal variables included were age, gender, pocket money, frequency of attendance at religious services, and risk perception of drug use scale. Family variables included were items regarding parental monitoring, parental smoking, and living with both parents.

**Table 1:** Descriptive features of Individual-level variables

Variable	(*n =* 45,273) %/mean	95 % CI
*Personal*		
Monthly smoking	39.9	39.5–40.4
Age (12–21)	15.5	15.4–15.5
Female	51.1	50.6–51.5
Educational level		
Grade 8	19.8	19,4–20–1
Grade 9	23.3	22.9–23.7
Grade 10	21.8	21.5–22–2
Grade 11	19.1	18.7–19.5
Grade 12	16.0	15.7–16.4
Pocket money		
Less than $US 10	30.0	29.5–30.4
$US 10–20	31.2	30.8–31.6
$US 20–40	21.4	21.0–21.8
$US 40–100	11.7	11.4–12.0
More than $US 100	5.7	5.5–5.9
How often do you attend religious services?		
Never or almost never	44.0	43.5–44.5
Once in a while monthly or yearly	34.3	33.8–34.7
Weekly	21.7	21.3–22.1
Risk perception on drug use	0,28	0–1
Lowest Q1, Q2, Q3	72.2	71.8–72.6
Highest Q1	27.8	27.4–28.2
*Family*		
With whom do you live?		
Both Mother and Father	67.3	66.8–67.7
Father and his partner	1.2	1.1–1.3
Mother and her partner	7.0	6.8–7.2
Only with your Father	2.3	2.2–2.4
Only with your Mother	18.4	18.1–18.8
With other person	3.8	3.7–4.0
Parental smoking		
No	44.7	44.2–45.2
Yes	55.3	54.8–55.8
Parental Monitoring		
How much are your parents aware of where you are after school?		
They never or almost never know where you are	5.9	5.7–6.1
Sometimes they know	25.3	24.9–25.7
They always or almost always know where you are	69.8	68.4–69.2
How much are your parents aware of what you do in school?		
Nothing	1.3	1.2–1.4
Some	15.3	15.0–15.6
Very Much	83.4	83.1–83.7
How well do your parents know your friends?		
A little	14.5	14.2–14.9
More or less	46.0	45.6–46.5
Very well	39.4	39.0–39.9
Having a talk with parents about drug risks		
Yes	68.5	68.1–68.9
No	31.5	31.1–31.9

**Table 2:** Descriptive features of school-level variables

Variable	Range	Mean/%	95 % CI
*School ethos*			
School bonding	5.9–11.5	8.46	8.45–8.46
School truancy	1.0–2.62	1.41	1.40–1.41
School drug availability perception	2.0–3.9	2.64	2.64–2.64
*School context*			
Math test score	174–355	256.13	255.79–256.46
School area			
Urban		97.9	97.7–98.0
Rural		2.1	2.0–2.3
School denomination			
Non-Religious		74.0	73.6–74.4
Religious		26.0	25.6–26.4
School Sex composition			
Only Girls		9.9	9.6–10.2
Mixed		83.7	83.4–84.1
Only boys		6.4	6.2–6.6
School type			
Municipal		40.9	40.4–41.3
Subsidized		51.9	51.4–52.3
Private		7.3	7.0–7.5

#### School-level variables (Level 2)

We organised these variable into two groups: i) school ethos, variables related to the surrounding ethos of students, built from answers from students attending the same school, and ii) school context, variables referring to contextual features of school gathered from the Chilean Ministry of Education, that is, independent from students’ perceptions (see Table 2 for descriptive features of school-level variables).

The school ethos variables from the SENDA’s 2007 survey were aggregated at the school level:

i) School bonding: a 3-item scale was used. Students were asked: i) “How much happy do you go to school?” (1 = Very unhappy to 5 = Very happy); ii) “Do you feel part of the school?” (1 = No and 2 = Yes); and iii) How would you describe the relationship with your teachers? It is…” (1 = Awful to 5 = Excellent). An exploratory factor analysis was performed, and the reliability was calculated. All three items were loaded in a single latent factor, and the alpha coefficient was 0.54. The individual school bonding score was calculated by adding the score for each item. The total score range was 3 to 12 (mean = 8.46 (Standard Deviation [SD], 1.73). Finally, this variable was aggregated at the school level for calculating the mean score for each school.
ii) School truancy: One item asking about individual truancy in the last 12 months was used. Possible answers were 1 = never to 4 = many times. The total score range was 1 to 4 (mean = 1.41; SD, 0.68). Finally, this variable was aggregated at the school level for calculating the mean score for each school.
iii) School drug perception: two items asking about having seen students in the school selling or using drugs were used. The total score range was 2 to 4 (mean = 2.64; SD, 0.82), and the alpha coefficient was 0.73. Finally, this variable was aggregated at the school level for calculating the mean score for each school.

School contextual variables from the Ministry of Education:

i) School math achievement: Each year, the Ministry of Education undertakes an assessment on math, language, natural science and social science subjects. All of these achievement results are highly correlated; therefore, only the math achievement result was used for this study to avoid co-linearity. This is a continuous variable and mean math score for each school was calculated using data from 2007 for 4th and 8th grade and from 2008 for 10th grade (range 174 to 355).
ii) School denomination: 0 = non-religious, 1 = religious.
iii) School sex composition: 0 = only girls; 1 = mixed; 2 = only boys.
iv) School type: 0 = municipal; 1 = subsidised; 2 = private. This variable can be considered as a *proxy* variable for the socio-economic status.
v) School location: 0 = urban and 1 = rural.

### Data analysis

After merging datasets, the final sample size was 45,273 students from 1,462 schools. The mean number of students per school was 31.

The main analysis was a multilevel logistic regression analysis. Multilevel analysis is recommended when data come from hierarchical levels. In this study, the students belonged to schools where they share context; therefore, we expected the same degree of similarity between their behaviours. Observations are not completely independent of one another [37, 38]. Multilevel logistic regressions allow examining the effect of individual-level and school-level or contextual factors on student behaviours [39, 40].

Smoking behaviour was the outcome variable, and it was treated as binary [41], based on whether the students smoked a cigarette any day during the last 30 days or not.

Different models were built. The null model was the reference and gave evidence of the existence of smoking prevalence variation between schools. Model 1 included all individual-level variables. Model 2 included all school-level variables. The final full model included all individual- and school-level variables that were associated with smoking in Models 1 and 2, at a significance level of *p*-value < 0.05. The fit for all models was assessed using the C-statistics, along with the 95 % CI, where a C-statistic of 1 is a perfect fit model and 0.5 is no better than chance [42]. A good fit model should have a C-statistic >0.7 [42].

Some cross-level interactions in the final model were explored such as sex and school test achievement and age and school bonding.

Stata 12.1 was used for all analyses.

## Results

### Sample description

The sample size was 45,273 adolescents attending 8th to 12th grade. Students were aged between 12 and 21 years with a mean age of 15.5 years (95 % CI, 15.4–15.5), and 51.1 % were female. Monthly smokers were 39.9 % of the students. One in five students attended religious services weekly. Most of the students had less than US $ 20 as pocket money. Most adolescents lived with their parents (67.3 %), and 55.3 % of students had at least one parent who smoked cigarettes. Regarding parental monitoring, most of the students said that their parents always or almost always knew where they were after school (69.8 %) and that they were very much aware of what students did at school (83.4 %). However, only 39.4 % of the parents knew their friends very well. Finally, 68.5 % of students reported that they had had a talk about drugs with their parents (See Table 1).

Most students attended urban, non-religious, mixed and subsidized schools. The mean school bonding score was 8.46 (range 5.9–11.5) (See Table 2).

### Multilevel analyses

#### Null model

The inter-school smoking variation was significant with an inter-correlation coefficient of 8.11 %.

#### Model 1: individual-level variables

The model had a good fit (C-statistic = 0.73). Most individual-level variables were associated with monthly smoking. Some of them increased the risk for smoking, such as being female, having a higher amount of pocket money, and parental smoking. Other factors decreased the likelihood of smoking, such us attending religious services on a weekly basis, living with both parents, and having higher parental monitoring. However, having a talk about drugs with parents was not related to smoking.

When compared to the null model, there was a reduction in the variance of smoking behaviour explained by schools (3.42 %).

#### Model 2: school factors

The model had a moderate fit (C-statistic = 0.65). Schools with higher school bonding reduced the risk for smoking, whereas schools with a higher level of truancy and student perception of drug availability increased the risk for smoking. School achievement had a clear effect on reducing the risk for smoking. It appears that schools where boys attend reduce the risk for smoking. Additionally, schools that receive students from high-income families (subsidized and private schools) had a higher risk for smoking. Finally, neither school location nor school denomination influenced the smoking behaviour among adolescents.

In this model, the variance based on school-level was reduced from 8.11 % (in the null model) to 3.39 %. This means that there was a reduction in approximately 58 % of the variance explained by the schools.

#### Full model

The model had a good fit (C-statistic = 0.73). When all school-level variables were controlled by individual-level variables, the same individual-level variables associated with smoking from Model 2 remained related to smoking. In terms of school-level variables, school bonding, school truancy, school drug availability perception and school math achievement remained associated with smoking behaviour.

The inter-class correlation coefficient was 2.46 %, that is, much of the variance explained by the school effect was due to the individual-level and school-level variables entered in the final model (See Table 3).

No significant interactions were found.

**Table 3:** Multilevel logistic regression modelling

	Null Model	Model 1 Individual-level	Model 2 School-level	Full Model
		OR (95 % CI)	OR (95 % CI)	OR (95 % CI)
***Individual-level***				
*Personal*				
Age		1.27 (1.25–1.29)		1.23 (1.21–1.25)
Gender				
Female		1		1
Male		0.65 (0.62–0.68)		0.64 (0.61–0.67)
Pocket money				
Less than $US 10		1		1
$US 10–20		1.22 (1,15–1.28)		1.22 (1.16–1.29)
$US 20–40		1.39 (1.31–1.47)		1.40 (1.32–1.48)
$US 40–100		1.68 (1.56–1.80)		1.69 (1.57–1.82)
More than $US 100		1.69 (1.54–1.86)		1.69 (1.53–1.85)
Religious service attendance				
Never or almost never		1		1
Once in a while monthly or yearly		0.97 (0.92–1.02)		0.98 (0.94–1.03)
Weekly		0.79 (0.74–0.83)		0.79 (0.74–0.84)
Risk perception on drug use				
Lowest Q1, Q2, Q3		1		1
Highest Q1		0.50 (0.48–0.52)		0.51 (0.48–0.53)
*Family*				
Parental smoking				
No		1		1
Yes		1.72 (1.65–1.80)		1.71 (1.64–1.78)
Family structure:				
Without both parents		1		1
With both parents		0.82 (0.78–0.86)		0.83 (0.79–0.87)
Parents know where they are after school				
They never or almost never know where you are		1		1
Sometimes they know		1.12 (1.02–1.23)		1.11 (1.02–1.22)
They always or almost always know where you are		0.55 (0.51–0.60)		0.56 (0.51–0.61)
Parents know what they do at school				
Nothing		1		1
Some		0.98 (0.81–1.18)		0.99 (0.82–1.20)
Very Much		0.82 (0.68–0.98)		0.83 (0.69–1.00)
Parents know friends				
A little		1		1
More or less		0.92 (0.87–0.98)		0.93 (0.88–0.99)
Very well		0.88 (0.83–0.94)		0.91 (0.85–0.97)
Talk about drug with parents				
No		1		1
Yes		0.99 (0.95–1.04)		0.99 (0.95–1.04)
***School-level***				
*School ethos*				
School Bonding			0.87 (0.82–0.92)	0.93 (0.88–0.98)
School Truancy			1.99 (1.74–2.27)	1.34 (1.18–1.53)
School Drug availability perception			1.76 (1.60–1.95)	1.23 (1.12–1.36)
*School context*				
Math Test score			0.997 (0.996–0.998)	0.997 (0.996–0.998)
School area				
Urban			1	1
Rural			0.95 (0.80–1.13)	0.99 (0.83–1.18)
School denomination				
Non-religious			1	1
Religious			1.05 (0.98–1.13)	1.06 (0.99–1.14)
School Sex composition				
Only girls			1	1
Co-educational			0.77 (0.69–0.84)	1.00 (0.91–1.11)
Only boys			0.67 (0.57–0.78)	1.02 (0.87–1.19)
School type (proxy variable of socioeconomic status)				
Municipal			1	1
Subsidized			1.22 (1.14–1.30)	1.06 (0.99–1.13)
Private			1.29 (1.13–1.47)	1.04 (0.92–1.19)
Random Intercept				
Beta (T00)	0,29*	0.34*	0.34*	0.29*
(Intra-class Correlation) ICC (%)	8.11	3.42	3.39	2.46
C-statistic (95%CI)		0.73 (0.73–0.74)	0.65 (0.64–0.65)	0.73 (0.72–0.73)

Note: * *p*-value < 0.001

## Discussion

### Main results

The last 30-day smoking prevalence in Chile found in this study was very high compared with other countries [29, 33, 43]. This confirmed previous findings that smoking among adolescents in Chile was among the highest worldwide. Therefore, it is very important to explore the factors associated to this behaviour.

This is the first Chilean study exploring the influence of school-related factors on the smoking behaviour of adolescents, controlling for individual variables. The main strengths of the study are the usage of a large nationally representative sample of students, the possibility of using some truly contextual factors, such as school achievement, and several other school-related factors potentially modifiable such as school bonding.

We found an inter-school variation on smoking behaviour similar to other studies [33, 44, 45]. In the null model, school level explained 8 % of the smoking behaviour. This means that even though most of the variance may be due to personal or other factors, a sizable proportion of this variance is explained at school level. From a policy-making point of view, this is important, considering the proportion of the population that attend schools and the potential impact that school interventions might have over and above the behaviour of adolescents.

The main school-level factors explaining the school influence on smoking are school bonding, school truancy, the perception of drug availability, and school math achievements. These findings are consistent with the idea that those schools more academically orientated, with better attendance, and schools where students feel more strongly bonded seem to provide a more protective environment or ethos against smoking behaviour. A recent review found that school ethos appears to be an important influence on adolescent smoking [24]. School bonding and school truancy had been previously considered as important modifiable individual-level protective factors against poor academic achievement [46], poor mental health [47] and substance misuse [48].

Even though, the influence of individual features on smoking behaviour was not the main aim of this study, it is worth mentioning that several well-known personal factors [7, 21] were also associated with smoking such as: older age, female sex, more pocket money, religious participation, lower drug risk perception, parental smoking, and low parental monitoring. We stress here the importance of the last three individual factors because these are potentially modifiable variables [49].

Overall our study provides additional evidence in support of the social capital theory, which postulates that healthy behaviours may be fostered by having good relationships between school personnel and students and positive ethos of stable and shared norms [50]. In addition our results are also in keeping with the social control theory, which explains that deviant behaviour may be reduced by increasing the sense of connectedness to a community [51].

It is possible to conceive school interventions that can aim to bring a more positive school ethos. The Child Development Project aimed to promote a sense of community and a climate of mutual respect and it led to less social dissatisfaction and social anxiety among young children [52]. However, it is still uncertain if these changes may impact on behaviours such as smoking or other substance misuse later in adolescence. An intervention focusing on a social developmental curriculum that promotes pro-social behaviours, including school and community components aiming to “rebuild the village” and create a “sense of ownership” [53], reduced drug use and school delinquency, but only among boys. These results suggest that variables closely related to the school bonding construct may be potentially modifiable and lead to a reduction in unhealthy behaviours. Moreover, the conclusions of a recent review about school environment interventions found that the few interventions that have been developed are “promising but [the evidence] is not definitive” [54].

### Limitations

The main limitation of this study is related to the cross-sectional design that makes difficult to establish causality. Another limitation is the use of retrospective, self-reported measures in an adolescent population which could have introduced some reporting bias related to comprehension of questions and decision-making issues [55] or retrieval errors (especially for long periods of time) [56], or students’ perceptions of confidentially of the information [57] or social desirability [58]. However, the questionnaires used in this survey have been extensively used in Chile, the main outcome referred to a brief time period (past 30 days); and plenty of measures were taken to ensure anonymity and confidentiality. Furthermore, there is evidence that the cognitive and situational factors mentioned above do not threaten the validity of self-reported measurements among students [59]. Some risk factors were not measured in this survey and could not be included in this study. For instance, depression and anxiety have been found to be related to smoking behaviour, but no information was gathered in this survey. Regarding school-related factors, school policies, exposure to anti-smoking preventive programs and teachers’ opinions about smoking are all missing.

## Conclusions

Some of the identified school-related factors are susceptible to modification. For instance, increasing school bonding and school attendance are strategies that have helped to improve other outcomes, such as academic achievement [60]. Therefore, interventions addressing these school factors may also help to reduce smoking and other substance use behaviours.

## Abbreviations

SENDA, National Service for the Prevention and Rehabilitation of Drugs and Alcohol abuse [Servicio Nacional para la Prevención de Drogas y Alcohol]; CONACE, National Council for Narcotics Control [Consejo Nacional Para el Control de Estupefacientes]; SIMCE, National System of the Measurement of the Quality of Education [Sistema de Medición de la Calidad de la Educación]; SD, Standard Deviation
